# Impact of Diabetic Foot on Selected Psychological or Social Characteristics

**DOI:** 10.1155/2014/981721

**Published:** 2014-10-21

**Authors:** Ugur Cakir, Ertugrul Kargi, Hakan Sarman, Cengiz Isik

**Affiliations:** ^1^Department of Psychiatry, Abant Izzet Baysal University, School of Medicine, Bolu, Turkey; ^2^Department of General Surgery, Abant Izzet Baysal University, School of Medicine, Bolu, Turkey; ^3^Department of Orthopaedics and Traumatology, Abant Izzet Baysal University, School of Medicine, Golkoy, 14280 Bolu, Turkey

With great interest, we read the recent paper [1] “*Does the diabetic foot have a significant impact on selected psychological or social characteristics of patients with diabetes mellitus*?”. The authors aimed to compare selected psychological and social characteristics between diabetic patients with and without the diabetic foot (DF). They have concluded that patients with DF had a predominantly worse standard of living and patients with DF appeared to have good stress tolerability and mental health and did not reveal severe forms of depression or any associated consequences.

It is very well known that comorbidity of depression with diabetes mellitus is related to poor glycemic control, higher severity of diabetic complications, increased risk of cardiovascular diseases, higher functional disability, and poor treatment adherence [2, 3].

In contradiction to what is mentioned above and the authors' expectation, the study results showed no significant differences between the two groups regarding depression scores. In addition to the authors' reasonable discussion, we want to stress another possible factor not mentioned in the study which is that the patients in both groups may be on or have history of antidepressant treatment. We believe it seems important to assess the presence of antidepressant treatment in the future studies to assess presence of depressive symptoms.
